# Association of acid phosphatase locus 1*C allele with the risk of cardiovascular events in rheumatoid arthritis patients

**DOI:** 10.1186/ar3401

**Published:** 2011-07-18

**Authors:** María Teruel, Jose-Ezequiel Martin, Carlos González-Juanatey, Raquel López-Mejias, Jose A Miranda-Filloy, Ricardo Blanco, Alejandro Balsa, Dora Pascual-Salcedo, Luis Rodriguez-Rodriguez, Benjamin Fernández-Gutierrez, Ana M Ortiz, Isidoro González-Alvaro, Carmen Gómez-Vaquero, Nunzio Bottini, Javier Llorca, Miguel A González-Gay, Javier Martin

**Affiliations:** 1 Instituto de Parasitología y Biomedicina "López-Neyra", IPBLN-CSIC, Avd. del Conocimiento s/n. 18010. Granada, Spain; 2 Division of Cardiology, Hospital Xeral-Calde, Dr Ochoa s/n, 27004, Lugo, Spain; 3 Rheumatology Division, Hospital Universitario Marqués de Valdecilla, IFIMAV, Avenida de Valdecilla s/n, 39008, Santander, Spain; 4 Rheumatology Division, Hospital Xeral-Calde, Dr Ochoa s/n, 27004, Lugo, Spain; 5 Rheumatology Service, Hospital Universitario La Paz, Paseo de la Castellana 261, 28046, Madrid, Spain; 6 Servicio de Reumatología, Hospital Clinico San Carlos, C/Profesor Martín Lagos, S/N, 28040 Madrid, Spain; 7 Rheumatology Service, Hospital Universitario La Princesa, C/Diego de León, 62,28006, Madrid, Spain; 8 Rheumatology Service, Hospital Universitari Bellvitge, Feixa Llarga s/n, 08907, Hospitalet de Llobregat, Barcelona, Spain; 9 Division of Cell Biology, La Jolla Institute for Allergy and Immunology, 9420 Athena Circle, La Jolla, CA 92037, USA; 10 Department of Epidemiology and Computational Biology, School of Medicine, University of Cantabria, and CIBER Epidemiología y Salud Pública (CIBERESP), IFIMAV, Avenida del Cardenal Herrera Oria, 39011, Santander, Spain

## Abstract

**Introduction:**

*Acid phosphatase locus 1 *(*ACP1*) encodes a low molecular weight phosphotyrosine phosphatase implicated in a number of different biological functions in the cell. The aim of this study was to determine the contribution of *ACP1 *polymorphisms to susceptibility to rheumatoid arthritis (RA), as well as the potential contribution of these polymorphisms to the increased risk of cardiovascular disease (CV) observed in RA patients.

**Methods:**

A set of 1,603 Spanish RA patients and 1,877 healthy controls were included in the study. Information related to the presence/absence of CV events was obtained from 1,284 of these participants. All individuals were genotyped for four *ACP1 *single-nucleotide polymorphisms (SNPs), rs10167992, rs11553742, rs7576247, and rs3828329, using a predesigned TaqMan SNP genotyping assay. Classical *ACP1 *alleles (*A, *B and *C) were imputed with SNP data.

**Results:**

No association between *ACP1 *gene polymorphisms and susceptibility to RA was observed. However, when RA patients were stratified according to the presence or absence of CV events, an association between rs11553742*T and CV events was found (*P *= 0.012, odds ratio (OR) = 2.62 (1.24 to 5.53)). Likewise, the ACP1*C allele showed evidence of association with CV events in patients with RA (*P *= 0.024, OR = 2.43).

**Conclusions:**

Our data show that the ACP1*C allele influences the risk of CV events in patients with RA.

## Introduction

Rheumatoid arthritis (RA) is a complex polygenic autoimmune inflammatory disease characterized by persistent synovitis and joint damage. Several genetic polymorphisms, such as *HLA-DRB1*, *PTPN22*, *STAT4*, *TRAF1/C5 *and *TNFAIP3*, have been implicated in the susceptibility to RA [1]. On the other hand, increased cardiovascular (CV) mortality is observed in patients with RA. This is the result of accelerated atherogenesis [2-4].

*Acid phosphatase locus *1 (*ACP1*) is a gene located on chromosome 2p25 that encodes a low molecular weight phosphotyrosine phosphatase (LMW-PTP), which presents two main enzymatic activities: phosphoprotein tyrosine phosphatase and flavin mononucleotide phosphatase [5]. Two different isoenzymes of LMW-PTP have been described: 'fast' (also noted as ACP1-F(fast), isoform 1, IF1, HCPTP-A) and 'slow' (also noted as ACP1-S(slow), isoform 2, IF2, HCPTP-B), that arise through alternative splicing mechanisms, in which either exon 3 or exon 4 is excised and the other retained respectively [5,6]. These two LMW-PTP isoenzymes have different molecular and catalytic properties, suggesting that they may be implicated in different biological functions in the cell [5,7]. In Caucasian populations there are three common codominant alleles of *ACP1*, ACP1*A, ACP1*B, ACP1*C. *ACP1 *alleles differ on single-nucleotide polymorphisms (SNPs), which affect both the total enzymatic activity and the ratio between isoforms F/S, being the ratio F/S 2:1 in ACP1*A, 4:1 in ACP1*B and 1:4 in ACP1*C [5,7,8].

LMW-PTP is considered to play a key role as regulator of signaling pathways in receptor-stimulated immune cells [9]. LMW-PTP has also been involved in the regulation of many growth factors such as platelet-derived growth factor receptor (PDGFR) [10], fibroblast growth factor receptor (FGFR) [11], insulin receptor (IR) [12,13] and EphA2 receptor, a ligand that binds to the Ephrin family of signaling molecules [14]. LMW-PTP has also been implicated in the regulation of ZAP70 Kinase (ζ-chain- associated protein kinase of 70 kDa) [15] playing a role in T-cell development and lymphocyte activation, enhancing signaling from the T cell antigen receptor [15]. Additionally, LMW-PTP has been found to be a key mediator in the integrin signaling during cellular adhesion [9].

Allelic polymorphisms of the *ACP1 *gene have been associated with susceptibility to several human diseases, including inflammatory and autoimmune diseases [5,16]. Interestingly, the *ACP1 *gene was also associated with susceptibility to coronary atherosclerotic artery disease (CAD) [17].

Taking into account the possible influence that *ACP1 *may have in the susceptibility to immune-mediated disorders and in the pathogenesis of the CV disease, in the present study we aimed to investigate the possible association of *ACP1 *alleles with the susceptibility to RA as well as whether *ACP1 *gene polymorphism may contribute to the increased risk of CV complications observed in patients with RA.

## Materials and methods

### Material

A set of 1,603 RA Spanish patients and 1,877 healthy individuals were included in the present study. Blood samples were obtained from RA patients recruited from the Hospital Xeral-Calde (Lugo), Hospital Universitario Marqués de Valdecilla (Santander), Hospital Universitario Bellvitge (Barcelona), and Hospital La Paz, Hospital de La Princesa and Hospital Clínico San Carlos (Madrid). All the patients fulfilled the 1987 American College of Rheumatology (ACR) criteria for the classification of RA [18].

Information related to the presence or absence of CV events was obtained in 1,284 RA patients (80.1%, 1284/1,603). Among them, 229 experienced CV events (17.8%, 229/1,284). Information on traditional CV risk factors was also collected.

Clinical features of the whole series of 1,603 RA patients are shown in Table 1.

**Table 1:** Demographic characteristics of the patients with rheumatoid arthritis included in the study

Clinical feature	% (n/N)
Patients	1,603
**Main characteristics**	
Age at disease onset (years, means ± SE)	54.1 ± 14.8
Follow up (years, means ± SE)	11 ± 7.5
Female	73.5
Rheumatoid factor positive	70.3 (996/1,417)
Shared Epitope positive	63.7 (592/930)
Anti-CCP antibodies positive	58.1 (652/1,123)
**Cardiovascular risk factors**	
Hypertension	39.4 (516/1,310)
Diabetes mellitus	13.2 (171/1,300)
Dyslipidemia	41.3 (540/1,307)
Obesity	12.4 (142/1,146)
Smoking habit	24.0 (303/1,261)
**Patients with cardiovascular events**	17.8 (229/1,284)
Ischemic heart disease	9.5 (122/1,284)
Heart failure	4.8 (62/1,284)
Cerebrovascular accidents	4.6 (59/1,284)
Peripheral arteriopathy	1.9 (25/1,284)

SE, Standard error

Anti-CCP antibodies, anti-cyclic citrullinated peptide antibodies

A CV event was considered to be present if the patient had ischemic heart disease, heart failure, a cerebrovascular accident or peripheral artheriopathy. Clinical definitions for CV events and classic CV risk factors were established as previously described [4,19]. The study was approved by local ethics committees from all the participating centers and all subjects provided informed consent according to the Declaration of Helsinki.

### SNPs selection and genotyping

DNA from patients and controls was obtained using standard methods. We selected four *ACP1 *SNPs for the present study. rs11553742 and rs7576247 were selected because of their ability to tag classical *ACP1 *alleles (that is, ACP1*A, ACP1*B, ACP1*C) [5]. rs11553742 is a synonymous polymorphism located in the codon 44 (exon 3) and rs7576247 encodes an aminoacid change in the codon 105 (exon 6) from arginine, present in ACP1*A allele, to glutamine in ACP1*B and *C alleles. Hence, ACP1*A allele differs from ACP1*C allele in two base substitutions in those positions, so the CG allele combination is responsible for the ACP1*A allele and TA for the ACP1*C allele. In addition, ACP1*B allele is defined as not *A, not *C, that is, for the allelic combination CA. Another two polymorphisms, rs10167992 and rs3828329, were also selected because they showed association with quantitative traits related to type 2 diabetes mellitus [17]. All SNPs were genotyped with TaqMan SNP genotyping assays in a 7900 HT Real-Time polymerase chain reaction (PCR) system, according to the conditions recommended by the manufacturer (Applied Biosystems, Foster City, CA, USA). All samples were genotyped at the same center.

### Statistical analysis

Controls were tested for significant differences in their genotype distribution and Hardy-Weinberg equilibrium (HWE) theoretical distribution by means of a χ^2 ^test. The case-control association study was performed by 2 × 2 contingency tables with χ^2 ^to obtain *P*-values, odds ratios (OR) and 95% confidence intervals (CI), according to Woolf's methods. The same procedure was applied in the subgroups stratified according to the presence or absence of anti-cyclic citrullinated peptide antibodies (ACPA). Association analysis for CV events in RA patients was performed via multiple logistic regression; estimates were adjusted for age at the time of disease diagnosis, gender, rheumatoid shared epitope status and traditional CV risk factors (hypertension, diabetes mellitus, dyslipidemia, obesity and smoking habit) as potential confounders.

All *P*-values < 0.05 were considered as statistically significant. All statistical analyses were carried out with Plink [20] and haplotype analysis with Haploview [21].

The estimation of the statistical power of the study to detect an effect of a polymorphism in disease susceptibility was performed using the CaTS Power Calculator software (Center for Statistical Genetics, University of Michigan, Michigan, USA) [22]. The study had between 98 and 100% power to detect the relative risk, with an OR of 1.50 at the 5% significance level, assuming a RA Spanish prevalence of this disease of 0.5% and considering a minor allele frequency (MAF) between 0.05 and 0.25 respectively. Under the same conditions described above, our study of the risk of CV events in RA patients had a statistical power from 95% when the disease allele frequency was 0.25 to 42% for an allele frequency of 0.05.

## Results

### *ACP1 *polymorphisms in RA patients and controls

All genetic variants analyzed did not deviate significantly from the HWE, and the genotyping success call rate was 90%. After comparing RA patients and healthy individuals, no significant differences in the *ACP1 *allele and genotype frequencies were found (Additional file 1). We also assessed the possible influence of these *ACP1 *polymorphisms in the presence and absence of ACPA; however, no evidence of association was observed. In addition, we performed the analysis of allelic combinations to investigate the possible association of each of these three codominant *ACP1 *alleles (*A, *B and *C) with RA but no significant association was found. Again, no association was observed for *ACP1 *alleles when RA patients were stratified according to ACPA (Additional file 2).

### *ACP1 *polymorphisms and CV risk in RA patients

We further investigated the possible influence of *ACP1 *polymorphisms in the risk of CV events in RA patients. Of the 1,284 RA patients for whom information on presence or absence of CV disease was available, 229 had CV events (17.8%). Table 2 describes the distribution of *ACP1 *polymorphisms in RA patients with and without CV events. After adjusting for classical CV risk factors, evidence of association of rs11553742*T with the risk of CV events was observed (*P*-adj = 0.012, OR = 2.62 (1.24 to 5.53)).

**Table 2:** Differences between RA patients with and without CV events according to *ACP1 *polymorphisms

	Change			Genotype, no. (frequency)			**Minor allele**,	Allele test	
SNP	1/2	Samples Set	N	1/1	1/2	2/2	no. (frequency)	*P*-adj*	OR (95% CI)*
rs10167992	C/T	RA with CV	215	171 (0.826)	35 (0.169)	1 (0.005)	37 (0.089)	0.321	0.72 (0.38 to 1.37)
		RA without CV	965	768 (0.799)	168 (0.175)	13 (0.014)	194 (0.102)		
rs11553742	C/T	RA with CV	221	200 (0.966)	21 (0.101)	0 (0.000)	21 (0.048)	0.012	2.62 (1.24 to 5.53)
		RA without CV	1,015	932 (0.970)	78 (0.081)	3 (0.003)	84 (0.041)		
rs7576247	A/G	RA with CV	207	112 (0.541)	76 (0.367)	18 (0.087)	112 (0.272)	0.203	0.76 (0.50 to 1.16)
		RA without CV	961	498 (0.518)	388 (0.404)	75 (0.078)	538 (0.280)		
rs3828329	C/T	RA with CV	221	88 (0.425)	103 (0.498)	28 (0.135)	641 (0.319)	0.079	1.38 (0.96 to 1.97)
		RA without CV	1,015	482 (0.502)	403 (0.419)	119 (0.124)	159 (0.363)		

CV, cardiovascular; RA, rheumatoid arthritis

* multiple regression adjusted by age at diagnosis of the disease, gender, shared epitope status and traditional CV risk factors, that is, hypertension, diabetes mellitus, dyslipidemia, obesity and smoking habit, as potential confounders.

The potential influence of ACP1*A, *B and *C alleles in the CV risk of RA patients was also analyzed (Table 3). We found that the ACP1*C allele was significantly associated with CV risk in RA patients after correction for classic CV risk factors (*P*-adj = 0.024, OR = 2.43). As expected, ACP1*C allele (TA) included the minor rs11553742*T allele, which was also found to be a risk factor for the CV events in RA patients (see Table 2).

**Table 3:** Distribution of *ACP1 *alleles in RA patients with and without CV events

		Haploype, no. (frequency)			
ACP1 allele	Haplotype	RA with CV	RA without CV	*P*-adj*	OR*
ACP1*A	CG	110 (0.276)	525 (0.281)	0.217	0.76
ACP1*B	CA	270 (0.678)	1,263 (0.676)	0.859	1.04
ACP1*C	TA	18 (0.045)	80 (0.043)	0.024	2.43

CV, cardiovascular; RA, rheumatoid arthritis The order of the SNPs is rs11553742|rs7576247.

* multiple regression adjusted by age at diagnosis of the disease, gender, shared epitope status, hypertension, diabetes mellitus, dyslipidemia, obesity and smoking habit.

In contrast, ACP1*A allele (CG), which was the opposite allelic combination of ACP*C, showed a trend for protection against the development of CV events in RA patients, although no statistically significant association was achieved (*P*-adj = 0.217, OR = 0.76).

## Discussion

Since the association of *ACP1 *gene with autoimmunity has previously been described [5], in the present study we sought to investigate the possible association of *ACP1 *polymorphisms with RA. Furthermore, taking into account that this gene has been involved in the susceptibility to CAD [17], we also assessed whether *ACP1 *variations could be involved in the risk of CV events in patients with RA. Our result revealed that *ACP1 *polymorphisms do not influence the susceptibility to RA. However, these polymorphisms seem to influence the risk of CV events in these patients. In this regard, both rs11553742*T and ACP1*C alleles increased the risk of CV complications in patients with RA. Interestingly, rs11553742*T has been observed to decrease the F/S ratio of the LMW-PTP isoenzymes [5]; in this regard the ACP1*C allele, carrier of the minor allele of rs11553742, was found to produce a major amount of S isoforms and is also associated with the highest total LMW-PTP activity [8,23].

Our results are in accordance with the findings by Banci *et al*. [17], who observed that high S isoform genotypes were associated with increased risk to develop CAD. Moreover, patients with hypertrophic cardiomyopathy, an autosomal dominant disease, were found to have the highest frequencies for ACP1*C allele and showed a linear relationship between maximum wall thickness and the amount of total LMW-PTP activity [16].

The effect of the ACP1*C allele in the development of CV events could be explained by its possible role in the regulation of the energy metabolism and oxidative stress through its flavin mononucleotide phosphatase activity [8]. With respect to this, a negative interaction between LMW-PTP and the enzyme glutathione reducatase (GSR), which affects the cellular concentration of their cofactor flavin adenosine dinucleotide (FAD), has been described [8]. GSR is a flavoenzyme involved in the cellular antioxidant mechanism that reduces oxidized glutathione disulfide (GSSG) to the sulfhydryl form glutathione (GSH) that is an important cellular antioxidant. Low LMW-PTP activity increases the levels of cofactor flavin adenine dinucleotide (FAD) in the cytosol leading to increased activity of GSR; while higher LMW-PTP activity yields low GSR activity. Accordingly, low activity of GSR has also been found to be significantly associated with hypertension [24], and it has also been considered to be a risk factor for CV by influencing cholesterol levels [25]. Furthermore, Bottini *et al*. [26] reported that the ACP1*A allele, the opposite allelic combination of ACP*C, is a protective factor for hypertriglyceridemia and hypercholesterolemia in obese women.

RA is a complex polygenic disease and, besides the association of HLA-DRB1*04 shared epitope alleles with CV disease [4,27], recent reports have also emphasized the potential implication of other gene polymorphisms in the increased risk of CV events observed in patients with RA. In this regard, interactions between *NOS *gene polymorphisms and HLA-DRB1*04 shared epitope alleles seem to confer an increased risk of developing CV events in these patients [28]. Also, the A1298C polymorphism in the *MTHFR *gene was found to predispose to CV risk in RA [29]. More recently, an association of the *TNFA *rs1800629 gene polymorphism with predisposition to CV complications in RA patients carrying the rheumatoid shared epitope was also described [30].

## Conclusions

Our data show for first time the association of the ACP1*C allele with increased susceptibility to CV events in patients with RA. This effect may be based on the major production of the S isoform of LMW-PTP by this allele, which may influence the regulation of energy metabolism and the response to oxidative stress.

## Abbreviations

ACP1: acid phosphatase locus 1; ACPA: anti-cyclic citrullinated peptide antibodies; ACR: American College of Rheumatology; CAD: coronary atherosclerotic artery disease; CI: confidence intervals; CV: cardiovascular; FAD: flavin adenosine dinucleotide; FGFR: fibroblast growth factor receptor; GSH: glutathione; GSR: glutathione reducatase; GSSR: glutathione disulfide; HWE: Hardy-Weinberg equilibrium; IR: insulin receptor; LMW-PTP: low molecular weight phosphotyrosine phosphatase; MAF: minor allele frequency; OR: Odds ratio; PCR: polymerase chain reaction; PDGFR: platelet-derived growth factor receptor; RA: rheumatoid arthritis; SNP: single-nucleotide polymorphism.

## Competing interests

The authors declare that they have no competing interests.

## Authors' contributions

MT, JEM, NB and JM made substantial contributions to the conception and design of the study, and the interpretation of data. MT carried out genotyping, analysis of data and drafted the manuscript. JEM carried out genotyping. CGJ, RLM, JAMF, RB, AB, DPS, LRR, BFG, AMO, IGA and CGV were involved in the acquisition of cardiovascular data in the different Spanish hospitals included in this study. JL carried out the analysis and interpretation of the data. JM and MAGG were involved in revising the manuscript and gave final approval of the version to be published.

## Supplementary Material

Additional file 1 **Genotype and allele distribution of *ACP1 *polymorphisms in Spanish RA patients and healthy subjects**. Supplementary table S1 shows the genotype and allele frequencies of *ACP1 *polymorphisms in Spanish RA patients and healthy controls. That table also shows the lack of association among cases and controls. Click here for file

Additional file 2 **Distribution of *ACP1 *alleles in Spanish RA patients and healthy controls**. Supplementary table S2 shows the frequencies of *ACP1 *alleles in Spanish RA patients and individuals controls. No association was observed. Click here for file

## References

[B1] Gregersen PK Susceptibility genes for rheumatoid arthritis-a rapidly expanding harvest Bull NYU Hosp Jt Dis 2010 68 179 182 20969549

[B2] Gonzalez-Gay MA Gonzalez-Juanatey C Martin J Rheumatoid arthritis: a disease associated with accelerated atherogenesis Semin Arthritis Rheum 2005 35 8 17 10.1016/j.semarthrit.2005.03.004 16084219

[B3] Full LE Ruisanchez C Monaco C The inextricable link between atherosclerosis and prototypical inflammatory diseases rheumatoid arthritis and systemic lupus erythematosus Arthritis Res Ther 2009 11 217 10.1186/ar2631 19435478 PMC2688172

[B4] Gonzalez-Gay MA Gonzalez-Juanatey C Lopez-Diaz MJ Pineiro A Garcia-Porrua C Miranda-Filloy JA Ollier WE Martin J Llorca J HLA-DRB1 and persistent chronic inflammation contribute to cardiovascular events and cardiovascular mortality in patients with rheumatoid arthritis Arthritis Rheum 2007 57 125 132 10.1002/art.22482 17266100

[B5] Bottini N Bottini E Gloria-Bottini F Mustelin T Low-molecular-weight protein tyrosine phosphatase and human disease: in search of biochemical mechanisms Arch Immunol Ther Exp (Warsz) 2002 50 95 104 12022706

[B6] Lazaruk KD Dissing J Sensabaugh GF Exon structure at the human ACP1 locus supports alternative splicing model for f and s isozyme generation Biochem Biophys Res Commun 1993 196 440 446 10.1006/bbrc.1993.2269 8216326

[B7] Dissing J Immunochemical characterization of human red cell acid phosphatase isozymes Biochem Genet 1987 25 901 918 10.1007/BF00502609 3130837

[B8] Apelt N da Silva AP Ferreira J Alho I Monteiro C Marinho C Teixeira P Sardinha L Laires MJ Mascarenhas MR Bicho MP ACP1 genotype, glutathione reductase activity, and riboflavin uptake affect cardiovascular risk in the obese Metabolism 2009 58 1415 1423 10.1016/j.metabol.2009.05.007 19570551

[B9] Souza AC Azoubel S Queiroz KC Peppelenbosch MP Ferreira CV From immune response to cancer: a spot on the low molecular weight protein tyrosine phosphatase Cell Mol Life Sci 2009 66 1140 1153 10.1007/s00018-008-8501-8 19002379 PMC11131502

[B10] Chiarugi P Cirri P Raugei G Manao G Taddei L Ramponi G Low M(r) phosphotyrosine protein phosphatase interacts with the PDGF receptor directly via its catalytic site Biochem Biophys Res Commun 1996 219 21 25 10.1006/bbrc.1996.0174 8619809

[B11] Rigacci S Rovida E Bagnoli S Dello Sbarba P Berti A Low M(r) phosphotyrosine protein phosphatase activity on fibroblast growth factor receptor is not associated with enzyme translocation FEBS Lett 1999 459 191 194 10.1016/S0014-5793(99)01234-X 10518016

[B12] Taddei ML Chiarugi P Cirri P Talini D Camici G Manao G Raugei G Ramponi G LMW-PTP exerts a differential regulation on PDGF- and insulin-mediated signaling Biochem Biophys Res Commun 2000 270 564 569 10.1006/bbrc.2000.2456 10753664

[B13] Pandey SK Yu XX Watts LM Michael MD Sloop KW Rivard AR Leedom TA Manchem VP Samadzadeh L McKay RA Monia BP Bhanot S Reduction of low molecular weight protein-tyrosine phosphatase expression improves hyperglycemia and insulin sensitivity in obese mice J Biol Chem 2007 282 14291 14299 10.1074/jbc.M609626200 17353188

[B14] Kikawa KD Vidale DR Van Etten RL Kinch MS Regulation of the EphA2 kinase by the low molecular weight tyrosine phosphatase induces transformation J Biol Chem 2002 277 39274 39279 10.1074/jbc.M207127200 12167657

[B15] Bottini N Stefanini L Williams S Alonso A Jascur T Abraham RT Couture C Mustelin T Activation of ZAP-70 through specific dephosphorylation at the inhibitory Tyr-292 by the low molecular weight phosphotyrosine phosphatase (LMPTP) J Biol Chem 2002 277 24220 24224 10.1074/jbc.M202885200 11976341

[B16] Bottini E Gloria-Bottini F Borgiani P ACP1 and human adaptability. 1. Association with common diseases: a case-control study Hum Genet 1995 96 629 637 10.1007/BF00210290 8522318

[B17] Banci M Saccucci P D'Annibale F Dofcaci A Trionfera G Magrini A Bottini N Bottini E Gloria-Bottini F ACP1 genetic polymorphism and coronary artery disease: an association study Cardiology 2009 113 236 242 10.1159/000203405 19246900

[B18] Arnett FC Edworthy SM Bloch DA McShane DJ Fries JF Cooper NS Healey LA Kaplan SR Liang MH Luthra HS The American Rheumatism Association 1987 revised criteria for the classification of rheumatoid arthritis Arthritis Rheum 1988 31 315 324 10.1002/art.1780310302 3358796

[B19] Gonzalez-Juanatey C Llorca J Martin J Gonzalez-Gay MA Carotid intima-media thickness predicts the development of cardiovascular events in patients with rheumatoid arthritis Semin Arthritis Rheum 2009 38 366 371 10.1016/j.semarthrit.2008.01.012 18336869

[B20] Purcell S Neale B Todd-Brown K Thomas L Ferreira MA Bender D Maller J Sklar P de Bakker PI Daly MJ Sham PC PLINK: a tool set for whole-genome association and population-based linkage analyses Am J Hum Genet 2007 81 559 575 10.1086/519795 17701901 PMC1950838

[B21] Barrett JC Fry B Maller J Daly MJ Haploview: analysis and visualization of LD and haplotype maps Bioinformatics 2005 21 263 265 10.1093/bioinformatics/bth457 15297300

[B22] Skol AD Scott LJ Abecasis GR Boehnke M Joint analysis is more efficient than replication-based analysis for two-stage genome-wide association studies Nat Genet 2006 38 209 213 10.1038/ng1706 16415888

[B23] Spencer N Hopkinson DA Harris H Quantitative differences and gene dosage in the human red cell acid phosphatase polymorphism Nature 1964 201 299 300 10.1038/201299a0 14110459

[B24] Chaves FJ Mansego ML Blesa S Gonzalez-Albert V Jimenez J Tormos MC Espinosa O Giner V Iradi A Saez G Redon J Inadequate cytoplasmic antioxidant enzymes response contributes to the oxidative stress in human hypertension Am J Hypertens 2007 20 62 69 10.1016/j.amjhyper.2006.06.006 17198913

[B25] Serdar Z Aslan K Dirican M Sarandol E Yesilbursa D Serdar A Lipid and protein oxidation and antioxidant status in patients with angiographically proven coronary artery disease Clin Biochem 2006 39 794 803 10.1016/j.clinbiochem.2006.02.004 16600205

[B26] Bottini N MacMurray J Peters W Rostamkhani M Comings DE Association of the acid phosphatase (ACP1) gene with triglyceride levels in obese women Mol Genet Metab 2002 77 226 229 10.1016/S1096-7192(02)00120-8 12409270

[B27] Farragher TM Goodson NJ Naseem H Silman AJ Thomson W Symmons D Barton A Association of the HLA-DRB1 gene with premature death, particularly from cardiovascular disease, in patients with rheumatoid arthritis and inflammatory polyarthritis Arthritis Rheum 2008 58 359 69 10.1002/art.23149 18240242 PMC3001034

[B28] Gonzalez-Gay MA Llorca J Palomino-Morales R Gomez-Acebo I Gonzalez-Juanatey C Martin J Influence of nitric oxide synthase gene polymorphisms on the risk of cardiovascular events in rheumatoid arthritis Clin Exp Rheumatol 2009 27 116 119 19327239

[B29] Palomino-Morales R Gonzalez-Juanatey C Vazquez-Rodriguez TR Rodriguez L Miranda-Filloy JA Fernandez-Gutierrez B Llorca J Martin J Gonzalez-Gay MA A1298C polymorphism in the MTHFR gene predisposes to cardiovascular risk in rheumatoid arthritis Arthritis Res Ther 2010 12 R71 20423475 10.1186/ar2989 PMC2888227

[B30] Rodríguez-Rodríguez L González-Juanatey C Palomino-Morales R Vázquez-Rodríguez TR Miranda-Filloy JA Fernández-Gutiérrez B Llorca J Martin J González-Gay MA TNFA -308 (rs1800629) polymorphism is associated with a higher risk of cardiovascular disease in patients with rheumatoid arthritis Atherosclerosis 2011 216 125 30 10.1016/j.atherosclerosis.2010.10.052 21420089

