# Supplementary material for: Association of acid phosphatase locus 1*C allele with the risk of cardiovascular events in rheumatoid arthritis patients
Source: Arthritis Res Ther. 2011 Jul 18;13(4):R116. doi: 10.1186/ar3401 (PMC3239354; doi:10.1186/ar3401)
Supplement: Additional file 1 — Genotype and allele distribution of ACP1 polymorphisms in Spanish RA patients and healthy subjects. Supplementary table S1 shows the genotype and allele frequencies of ACP1 polymorphisms in Spanish RA patients and healthy controls. That table also shows the lack of association among cases and controls. [file ar3401-S1.DOC]

**Additional file 1**

**Supplementary table S1**. Genotype and allele distribution of *ACP1* polymorphisms in Spanish RA patients and healthy subjects.

|  | Change |  |  | Genotype, no. (frequency) | | | Minor allele, | Allele test | |
| --- | --- | --- | --- | --- | --- | --- | --- | --- | --- |
| SNP | 1/2 | Samples Set | N | 1/1 | 1/2 | 2/2 | no. (frequency) | P-value | OR [95 % CI] |
| rs10167992 | C/T | Controls | 1729 | 1409 (0.815) | 300 (0.174) | 20 (0.012) | 340 (0.098) |  |  |
|  |  | RA | 1419 | 1149 (0.810) | 256 (0.180) | 14 (0.010) | 284 (0.100) | 0.817 | 1.02 [0.86-1.20] |
|  |  | RA-ACPA + | 558 | 448 (0.803) | 105 (0.188) | 5 (0.009) | 115 (0.103) | 0.647 | 1.05 [0.84-1.32] |
|  |  | RA-ACPA – | 420 | 347 (0.826) | 66 (0.157) | 7 (0.017) | 80 (0.095) | 0.787 | 0.97 [0.75-1.25] |
| rs11553742 | C/T | Controls | 1738 | 1574 (0.906) | 160 (0.092) | 4 (0.002) | 168 (0.048) |  |  |
|  |  | RA | 1539 | 1413 (0.918) | 120 (0.078) | 6 (0.004) | 132 (0.043) | 0.292 | 0.88 [0.70-1.11] |
|  |  | RA-ACPA + | 626 | 576 (0.920) | 48 (0.077) | 2 (0.003) | 52 (0.042) | 0.328 | 0.85 [0.62-1.17] |
|  |  | RA-ACPA – | 452 | 419 (0.927) | 33 (0.073) | 0 (0.000) | 33 (0.037) | 0.130 | 0.75 [0.51-1.09] |
| rs7576247 | A/G | Controls | 1773 | 932 (0.526) | 708 (0.399) | 133 (0.075) | 974 (0.275) |  |  |
|  |  | RA | 1451 | 768 (0.529) | 568 (0.391) | 115 (0.079) | 798 (0.275) | 0.978 | 1.00 [0.90-1.12] |
|  |  | RA-ACPA + | 572 | 309 (0.540) | 225 (0.393) | 38 (0.066) | 301 (0.263) | 0.445 | 0.94 [0.81-1.10] |
|  |  | RA-ACPA – | 430 | 228 (0.530) | 163 (0.379) | 39 (0.091) | 241 (0.280) | 0.744 | 1.03 [0.87-1.21] |
| rs3828329 | C/T | Controls | 1720 | 772 (0.449) | 731 (0.425) | 217 (0.126) | 1165 (0.339) |  |  |
|  |  | RA | 1522 | 709 (0.466) | 632 (0.415) | 181 (0.119) | 994 (0.327) | 0.301 | 0.95 [0.85-1.05] |
|  |  | RA-ACPA + | 623 | 284 (0.456) | 263 (0.422) | 76 (0.122) | 415 (0.333) | 0.720 | 0.98 [0.85-1.12] |
|  |  | RA-ACPA – | 442 | 208 (0.471) | 181 (0.410) | 53 (0.120) | 287 (0.325) | 0.432 | 0.94 [0.80-1.10] |

RA: Rheumatoid arthritis. ACPA: anti-cyclic citrullinated peptide antibodies. +, positive; –: negative.
