# Supplementary material for: Association of acid phosphatase locus 1*C allele with the risk of cardiovascular events in rheumatoid arthritis patients
Source: Arthritis Res Ther. 2011 Jul 18;13(4):R116. doi: 10.1186/ar3401 (PMC3239354; doi:10.1186/ar3401)
Supplement: Additional file 2 — Distribution of ACP1 alleles in Spanish RA patients and healthy controls. Supplementary table S2 shows the frequencies of ACP1 alleles in Spanish RA patients and individuals controls. No association was observed. [file ar3401-S2.DOC]

**Additional file 2**

**Supplementary table S2**. Distribution of *ACP1* alleles in Spanish RA patients and healthy controls.

|  |  |  | Haplotype, |  |  |
| --- | --- | --- | --- | --- | --- |
| ACP1 alleles | Haplotype | Samples Set | no. (frequency) | P-value | OR [95 % CI] |
| ACP1*A | CG | Controls | 911 (0.273) |  |  |
|  |  | RA | 771 (0.276) | 0.829 | 1.01 [0.90-1.13] |
|  |  | RA-ACPA + | 292 (0.263) | 0.550 | 0.96 [0.82-1.11] |
|  |  | RA-ACPA – | 237 (0.282) | 0.589 | 1.05 [0.89-1.24] |
| ACP1*B | CA | Controls | 2264 (0.677) |  |  |
|  |  | RA | 1909 (0.682) | 0.762 | 1.02 [0.91-1.13] |
|  |  | RA-ACPA + | 762 (0.687) | 0.542 | 1.05 [0.90-1.21] |
|  |  | RA-ACPA – | 567 (0.674) | 0.866 | 0.98 [0.84-1.16] |
| ACP1*C | TA | Controls | 161 (0.048) |  |  |
|  |  | RA | 118 (0.042) | 0.254 | 0.87 [0.68-1.11] |
|  |  | RA-ACPA + | 48 (0.043) | 0.506 | 0.91 [0.65-1.26] |
|  |  | RA-ACPA – | 30 (0.036) | 0.121 | 0.75 [0.51-1.11] |

RA: Rheumatoid arthritis. ACPA: anti-cyclic citrullinated peptide antibodies. +, positive; –: negative.

The order of the SNPs is rs11553742|rs7576247.
